# Can Platelet Indices Be New Biomarkers for Severe Endometriosis?

**DOI:** 10.1155/2014/713542

**Published:** 2014-03-26

**Authors:** Sümeyra Nergiz Avcioğlu, Sündüz Özlem Altinkaya, Mert Küçük, Selda Demircan-Sezer, Hasan Yüksel

**Affiliations:** ^1^Department of Gynecology and Obstetrics, Adnan Menderes University, School of Medicine, 09100 Aydın, Turkey; ^2^Department of Gynecology and Obstetrics, Muğla Sıtkı Koçman University, School of Medicine, 48000 Muğla, Turkey

## Abstract

*Objective*. The aim of this study was to investigate whether platelet indices-mean platelet volume (MPV), platelet distribution width (PDW), and plateletcrit (PCT) would be useful as noninvasive biomarkers for determining the severity of endometriosis. *Methods*. A retrospective review of the medical charts of 164 patients diagnosed with endometriosis and who were operated on between 2001 and 2013 was performed. The stage of endometriosis was determined according to revised American Society for Reproductive Medicine criteria. *Results*. In patients with advanced endometriosis (Stages 3-4), PLT, PCT levels were found to be significantly higher and MPV, PDW values to be significantly lower when compared to initial endometriosis (Stages 1-2). In addition, there was a significant positive correlation between PLT (*r*: 0.800, *P*: 0.001) and PCT (*r*: 0.727, *P*: 0.002) and the inflammatory marker white blood cell count (WBC). *Conclusion*. Our finding may not sufficient for employing platelet indices solely in this differential diagnosis, but our finding could provide a suggestion for clinical physicians so that attention is paid to the value of platelet indices and that these may be taken into account when making decisions about the initial or advanced stages of endometriosis.

## 1. Introduction

Endometriosis is one of the most important benign chronic diseases affecting 6–10% of women of reproductive age, being mainly associated with pelvic pain, adhesion formation, and infertility. Endometriosis is characterized by the ectopic presence of endometrial stroma and epithelium [1, 2]. It can be classified into four stages: minimal, mild, moderate, and severe [3]. More advanced endometriosis can be deeply invasive or can present as ovarian endometriotic cysts (endometrioma) [4].

Many theories have been set forth about endometriosis. The implantation theory of Sampson, the coelomic metaplasia theory of Mayer, and the theory of induction constitute the three classic theories that attempt to explain the origin of endometriosis [5, 6]. However, classic theories have failed to establish a definitive pathogenetic mechanism for endometriosis. Recent studies addressing the matter of genetic predisposition have reported that genetic abnormalities may contribute to the development of endometriosis [7].

Besides genetics, growing evidence indicates the significant role played by immunological and inflammatory factors in the development of endometriosis [8]. In particular, chronic pelvic inflammation is a hallmark of endometriosis pathophysiology and thus inflammation is a factor that favors the development of endometriosis [9]. Also, evidence available to date indicates that immune and inflammatory factors, whether they are released by immune or peritoneal, endometrial or endometriotic cells, may play a critical role in ectopic survival, implantation, and growth of endometrial tissue [2, 10–13].

The gold standard for the diagnosis of endometriosis is laparoscopic inspection, ideally combined with histological confirmation [14]. However, instead of these invasive methods, there have been pursuits to standardize noninvasive tests for diagnosing and identifying the severity of endometriosis. Recent studies have revealed an association between platelet indices (plateletcrit (PCT), platelet distribution width (PDW), and mean platelet volume (MPV)) and the white blood cell (WBC) count and C-reactive protein (CRP), underlining the wide relationship between platelets and inflammation [15]. To the best of our knowledge, however, there is no data to be found about the relationship between these platelet indices and endometriosis. The primary objective of the present study was to determine whether PDW, PCT, and MPV would be useful markers in the assessment of the severity of endometriosis and whether they are correlated with inflammatory parameters like WBC.

## 2. Material and Methods

The present study was approved by the local ethics committee of Adnan Menderes University School of Medicine, where the study was conducted.

A retrospective review of medical charts of 164 patients diagnosed with endometriosis and who were operated on between January 2001 and June 2013 was performed. These represented all patients diagnosed and operated due to endometriosis in clinic. Endometriosis was diagnosed by laparoscopy or laparotomy and confirmed by clinical, radiological, operative, and histopathological examination, if it could be performed, in all patients. Records of operations that noted the presence or absence of endometriosis and the stage of disease according to the revised American Society for Reproductive Medicine (ASRM) criteria were included. Staging was based on the scoring for superficial or deep involvement of the peritoneum and/or ovary with endometriosis and on the degree of adhesion enclosure in the ovaries and/or tubes. The revised ASRM classification score was based on implants and adhesions separately, uterine position (anteflexion and retroflexion), peritoneal lesions of the anterior or posterior cul-de-sac, diameters of right and left ovarian endometriomas, and rectovaginal implants. Most women have minimal or mild endometriosis, which is characterized by superficial implants and mild adhesions. Moderate and severe endometriosis is characterized by chocolate cysts and more severe adhesions [16]. Total scores of 1–5 constituted stage 1 (minimal) disease, 6–15 stage 2 (mild), 16–40 stage 3 (moderate), and ≥40 stage 4 (severe). On the other hand, the demographic and laboratory parameters of patients were obtained from the medical charts. Hematological parameters, which consisted of the WBC, PLT, MPV, PDW, and the PCT, were analyzed. All complete blood count (CBC) analyses were performed in the hematology laboratory of our hospital. CBC analysis was performed with the same analyzer within 2 hours after the collection of blood samples using a Mindray BC 6800 analyzer (M68 LH LYSE, China).

### 2.1. Statistical Analysis

Student's *t*-tests and Mann-Whitney *U* tests were performed to compare study variables between patients with initial and advanced endometriosis. Pearson's correlation test was also computed to quantify associations between platelet indices (PLT, PDW, PCT, and MPV) and WBC in the study group. Statistical analysis was performed using the Statistical Package for Social Sciences (SPSS Inc., Chicago, IL, USA) statistical software for Windows (Version 18.0). *P* < 0.05 was regarded as statistically significant.

## 3. Results

A total of 164 patients were included in the current study. The mean age of the participants was 33.7 ± 7.7. Endometriosis was confirmed laparoscopically in a group of 94 (57.3%) women and by laparotomy in 69 (47.1%) women. Vaginal cyst aspiration was performed only in one patient (0.6%). The main complaint was chronic pelvic pain in 84 (51.2%) patients, primary infertility in 38 (23.2%) patients, secondary infertility in 9 (5.5%) patients, dysmenorrhea in 8 (4.9%) patients, and menometrorrhagia in 17 (10.4%) patients. In eight (4.9%) of the patients, endometriosis was determined incidentally. During the operations, ovarian chocolate cyst was not observed in 28 (17.1%) patients. On the other hand, 83 (50.6%) patients had unilateral and 53 (32.3%) patients had bilateral endometriomas. The revised ASRM (1996) staging was applied to all patients with endometriosis in the study. There were 40 (24.4%) patients in the initial stages (I and II) and 124 (75.6%) patients in advanced stages (III and IV). Platelet indices in both groups of patients are given in Table 1.

**Figure 1:**
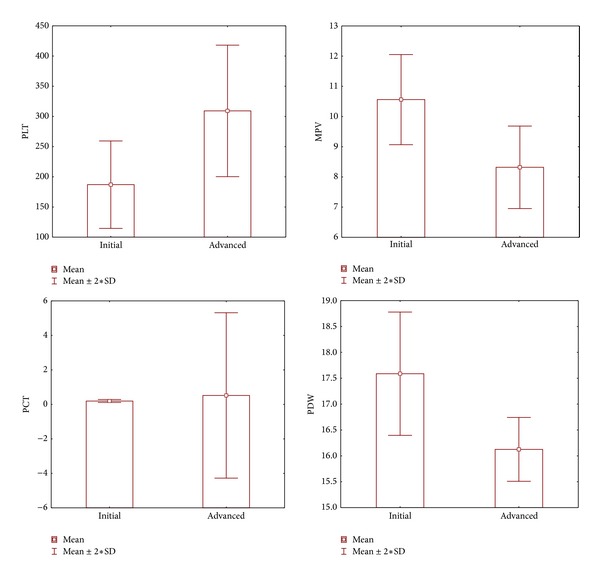
Correlations of platelet indices with initial and advanced stage of endometriosis. Plt: platelet (×10^9^/L), PCT: plateletcrit (%), PDW: platelet distribution width (fL), MPV: mean platelet volume (fL), and WBC: white blood cell (×10^9^/L); correlation is significant at the 0.05 level.

Hematological parameters consisted of the WBC range 4.5–10.3 × 10^9^/L, the PLT count range 156–373 × 10^9^/L, the MPV range 7.4–10.4 fL, the PDW range 15.6–18.2 fL, and the PCT range 0.155–0.320%. In patients with advanced endometriosis (Stages 3–4), MPV, PDW values were found to be significantly lower and PLT, PCT levels were found significantly higher when compared to initial endometriosis (Stages 1–2) (see Figures 1(a)–1(d)). In addition, there was a significant positive correlation between PLT (*r*: 0.800, *P*: 0.001) and PCT (*r*: 0.727, *P*: 0.002) and the WBC, which was an inflammatory marker blamed for the pathogenesis of endometriosis. Moreover, a significant negative correlation was determined between MPV (*r*: −0.738,  *P*: 0.001) and PDW (*r*: −0.724, *P*: 0.001) and the WBC count.

## 4. Discussion

In the present study, PLT and PCT values were found to be higher but PDW and MPV were determined to be lower in advanced endometriosis. Furthermore, PLT (*r*: 0.800, *P*: 0.001) and PCT (*r*: 0.727, *P*: 0.002) were positively and MPV (*r*: −0.738,  *P*: 0.001) and PDW (*r*: −0.724, *P*: 0.001) were inversely correlated with the WBC count, which was an inflammatory marker.

There are many studies in the literature about the development of endometriosis. Although endometriosis pathogenesis is still unclear, it has been proven to be both an estrogen-dependent and a chronic inflammatory disease [2, 17]. Elevated serum and peritoneal fluid inflammatory markers have been observed in several studies [18–20]. Pelvic pain, a rather frequent symptom of endometriosis, is relieved by anti-inflammatory drugs, supporting the contribution of chronic inflammation to the pathogenesis of this disease [21, 22]. Inflammation is blamed for the pathogenesis of endometriosis and occurs via many pathways. First of all, impaired immune response suppression and inadequate fibrinolytic mechanisms may mediate endometriosis pathogenesis. Moreover, variations in genes responsible for steroid response, inflammation, and tumor suppression may also contribute to the occurrence of the disease. Lastly, oxidative stress, exceeding the neutralizing capacity of the natural antioxidant mechanisms, may also trigger endometriosis [23]. It is known that the stress response causes the release of many hormones such as corticotrophin-releasing hormone, aggravating the inflammatory response in peritoneal lymphocytes [24].

Many studies on the role of other biomarkers in endometriosis (e.g., TNF-*α*, IL-6, VEGF, and CRP) have been conducted recently. The concentrations of various cytokines, growth and angiogenic factors, metalloproteinases, peptides, and subpopulations of leukocytes and the expression of various genes have been examined in endometriosis [25–27]. Nowadays researchers are trying to develop a statistical model based on three or four inexpensive, easily available serum biomarkers that could have enough statistical power to diagnose the severity of endometriosis without necessitating the expensive, invasive operative procedure of laparoscopy.

There have been many researches in the literature about the use of platelet indices in many conditions such as inflammatory bowel disease, Crohn's disease, and obesity, as well as cardiovascular and cerebrovascular diseases [28]. To the best of our knowledge, however, there has been no investigation about platelet indices in endometriosis. In the current study, we investigated these indices, which constitute inexpensive and easily measurable tests.

Platelets play a well-recognized role in hemostasis. Apart from this, there is increasing recognition of the importance of platelets during inflammatory processes. Upon activation, platelets release the contents of their *α*-granules, secreting a variety of cytokines, chemokines, and growth factors [29]. In stress conditions like inflammation, however, a positive correlation between thrombopoietin, ploidy of platelet progenitors, functional activity, and high platelet count is more apparent [30]. This is correlated with the current study as the platelet count was determined to be higher in severe endometriosis, which is claimed to be a high-grade inflammatory process.

Under normal conditions, the regulation of megakaryocytopoiesis is programmed to meet demands for activated platelets in physiological and pathological conditions, with resulting changes in platelet indices [31]. The interpretation of these resultant changes in platelet indices, however, is not always straightforward, considering the complexity of the inflammatory and immune mechanisms involved. The frequently described inverse relationship between platelet count and MPV in physiological and some pathological conditions reflects the tendency to maintain hemostasis by preserving a constant platelet mass [32]. This inverse relationship is often seen in especially high-grade systemic inflammation, where enhanced thrombopoiesis increases the quantity of circulating platelets, and large amounts of highly reactive large-sized platelets migrate to inflammatory sites, where they are intensely consumed [33]. In these conditions, MPV values decrease. Correlated with this knowledge, in the current study, MPV values were found to be lower in the advanced stages of endometriosis. This finding again supported the great role of inflammation in the pathogenesis of endometriosis.

PDW and PCT are often forgotten platelet indices and clinicians pay less attention to these than to platelet count and MPV. During the last decade, PDW and PCT have been evaluated in various studies on different disorders such as coronary artery disease, diabetes mellitus, pulmonary tuberculosis, obstructive sleep apnea, and inflammation [34–36]. PCT is a measurement derived from the platelet count and the MP. It has been accepted as an indicator of circulating platelets in a unit volume of blood [37]. PDW is a direct flow cytometric measurement of platelet cell volume [38]. In this study, PCT values were found to be higher and PDW values were lower in advanced endometriosis. Also, PDW and MPV values were inversely while PCT values were directly correlated with the inflammatory marker, WBC. These changes in platelet indices were supposed to have occurred due to reactive thrombocytosis in inflammation, which is blamed for the pathogenesis of endometriosis.

In conclusion, although the gold standard for the diagnosis of endometriosis is laparoscopic inspection, ideally combined with histological confirmation [14], the platelet count and platelet indices are tests that are rapidly available and easily obtained. Thrombocytosis is apparently a marker of chronic inflammation, which is blamed for the pathogenesis of endometriosis. Changes in platelet indices in endometriosis are also noteworthy and can be added especially to other inflammatory markers for determining the severity of disease. To the best of our knowledge, our study has for the first time discovered a preliminary but inspiring finding that MPV and other platelet indices could be used as good biomarkers in distinguishing initial and advanced endometriosis. We are aware that our finding is not sufficient for employing platelet indices solely in this differential diagnosis, but our finding could provide a suggestion for clinical physicians so that attention is paid to the value of platelet indices and that these may be taken into account when making decisions about the initial or advanced stages of endometriosis. Large, multicentered, prospective research on patients with endometriosis is needed, especially studies in which sensitivity and specificity analyses of platelet indices are carried out.

## Figures and Tables

**Table 1:** Inflammatory markers and platelet indices in patients with endometriosis.

Inflammatory markers & platelet indices	Initial endometriosis (*n* = 40)	Advanced endometriosis (*n* = 124)	*P*
Hgb^1^ (g/dL)	11.79 ± 1.79	11.62 ± 1.50	0.57
Hct^2^ (%)	35.33 ± 4.34	34.08 ± 3.77	0.082
WBC^3^ (×10^9^/L)	6.75 ± 1.29	11.61 ± 2.71	0.001
Plt^4^ (×10^9^/L)	187 ± 36.18	309.15 ± 54.43	0.001
MPV^5^ (fL)	10.56 ± 0.74	8.31 ± 0.68	0.003
PCT^6^ (%)	0.19 ± 0.43	0.30 ± 0.30	0.001
PDW^7^ (fL)	17.58 ± 0.59	16.12 ± 0.30	0.002

^1^Hgb: hemoglobin, ^2^Hct: hematocrit, ^3^WBC: white blood cell, ^4^Plt: platelet, ^5^MPV: mean platelet volume, ^6^PCT: plateletcrit, and ^7^PDW: platelet distribution width.
